# De Novo Mutation of m.3243A>G together with m.16093T>C Associated with Atypical Clinical Features in a Pedigree with MIDD Syndrome

**DOI:** 10.1155/2019/5184647

**Published:** 2019-04-04

**Authors:** Zhixin Jiang, Yinan Zhang, Jingbin Yan, Fengwen Li, Xinqian Geng, Huijuan Lu, Xiaoer Wei, Yanmei Feng, Congrong Wang, Weiping Jia

**Affiliations:** ^1^Shanghai Jiao Tong University Affiliated Sixth People's Hospital, Shanghai Key Laboratory of Diabetes, The Metabolic Diseases Biobank, Department of Endocrinology and Metabolism, Shanghai 200233, China; ^2^Shanghai Jiao Tong University Affiliated Sixth People's Hospital, The Metabolic Diseases Biobank, Center for Translational Medicine, Shanghai Key Laboratory of Diabetes, Shanghai 200233, China; ^3^Shanghai Institute of Medical Genetics, Shanghai Children's Hospital, Shanghai Jiao Tong University, The Key Laboratory of Embryo Molecular Biology, Ministry of Health of China & Shanghai Key Laboratory of Embryo and Reproduction Engineering, Shanghai 200040, China; ^4^Shanghai Jiao Tong University Affiliated Sixth People's Hospital, Department of Diagnostic Radiology, Shanghai 200233, China; ^5^Shanghai Jiao Tong University Affiliated Sixth People's Hospital, Department of Otolaryngology Head and Neck Surgery, Shanghai 200233, China

## Abstract

**Background:**

The syndrome of maternally inherited diabetes and deafness (MIDD) is typically caused by the m.3243A>G mutation and widely considered maternally inherited. In our study, we aimed to investigate the heredity way of the m.3243A>G among pedigrees with MIDD and discover novel mitochondrial DNA mutations related to atypical clinical phenotypes.

**Methods:**

Heteroplasmy levels of the m.3243A>G mutation in peripheral blood, saliva, and urine sediment of 31 individuals from 10 unrelated pedigrees were measured by pyrosequencing. Clinical evaluations including endocrinological, audiological, and magnetic resonance imaging (MRI) examinations, mitochondrial function evaluation in peripheral blood mononuclear cells (PBMCs), and whole mitochondrial DNA (mtDNA) sequencing were performed among the spontaneous mutant pedigrees.

**Results:**

Among the 10 unrelated MIDD pedigrees, we found that the de novo m.3243A>G mutation occurred in the family 1957 (F1957). The proband (F1957-II-1) and her son (F1957-III-1) both manifested diabetes with mild bilateral sensorineural hearing loss (SNHL) and abnormal brain MRI, and F1957-III-1 also complained of severe nausea and vomiting. Mitochondrial function evaluation in PBMCs revealed an increased level of ROS generation and decreased levels of ATP and mitochondrial membrane potential (ΔΨm) in the two m.3243A>G carriers. Whole mtDNA sequencing also revealed a de novo heteroplasmic substitution at m.16093T>C in both the proband and her son.

**Conclusions:**

Our study showed that de novo m.3243A>G mutation accompanied by other point mutations may occur in the very early embryonic or germ cell stage without maternal inheritance, bringing about both typical and atypical clinical features.

## 1. Introduction

The syndrome of maternally inherited diabetes and deafness (MIDD) is largely caused by an A-to-G transition at position 3243 of mitochondrial DNA (mtDNA) tRNALeu-encoding (UUR) gene, which is one of the most common point mutations of mtDNA [1]. The m.3243A>G mutation affects the tertiary structure of mitochondrial tRNALeu and leads to abnormal tRNA taurine modification and aminoacylation, thus resulting in a disorder of protein synthesis and cellular energy deficiency [2]. This syndrome usually affects metabolically active organs (such as endocrine pancreas and cochlea) and is accompanied by a wide range of clinical features including diabetes mellitus, sensorineural deafness, myopathy, congestive heart failure, and mitochondrial encephalomyopathy with lactic acidosis and stroke-like episodes (MELAS) [3].

Mitochondrial mutation is considered to be maternally inherited in most cases due to the fact that mitochondria in sperm are selectively destroyed and eliminated after fertilization, and only the mammalian mitochondria can pass on to the next generation. However, previous studies also suggest that spontaneous mutations may occur in families of mitochondrial disease, though the mechanism remains obscure [4–7].

The clinical manifestation associated with MIDD and MELAS may be heterogeneous. Although some aspects of the phenotype might be explained through the correlation of the heteroplasmy level of mutant mtDNA with disease severity [8–10], other regulatory factors, such as additional mitochondrial DNA mutations, are also found to influence clinical characteristics together with the m.3243A>G mutation [11–15].

We analyzed the heredity way of m.3243A>G among 10 pedigrees with MIDD and reported the family as having a spontaneous mutation during oogenesis or embryogenesis. Clinical characteristics and mitochondrial function in peripheral blood mononuclear cells (PBMCs) within the family were detailed analyzed. In addition, whole mtDNA sequencing was also performed to clarify the relationship between mtDNA sequence variations and atypical clinical phenotypes in the pedigree with de novo m.3243A>G mutation.

## 2. Materials and Methods

### 2.1. Study Population

In this study, a total of 31 individuals with m.3243A>G mutation from 10 unrelated pedigrees were enrolled from the Shanghai Clinical Medical Center of Diabetes. The study was approved by the Institutional Review Board of Shanghai Jiao Tong University Affiliated Sixth People's Hospital and was conducted in accordance with the Declaration of Helsinki. Written informed consent was obtained from each subject. Standard questionnaires were conducted for each participant to obtain their general information.

### 2.2. m.3243A>G Mutation Analysis

Peripheral blood, saliva, and urine sediments were obtained from all participants. Urinary sediments were collected from 10 ml of the fresh urine voided by each participant after centrifugation and then washed by phosphate-buffered saline (PBS) twice. Cellular DNA was extracted from respective samples using an automated nucleic acid extraction instrument (Lab-Aid 820; BioV, China). Pyrosequencing was used to determine the accurate quantification of the heteroplasmy levels of m.3243A>G mutation as previously described [16].

### 2.3. Clinical Evaluation among the Family 1957 (F1957)

Biochemical examinations were performed for individuals in F1957 including routine blood and urine tests. Fasting and 2 h postprandial plasma glucose, glycated hemoglobin (HbA1c), glycated albumin (GA), and fasting and 2 h postprandial C-peptide were determined for routine diabetes screening. Antibodies against glutamic acid decarboxylase (GAD) and islet antigen 2 (IA2) were tested to exclude type 1 diabetes. Blood lactic acid was analyzed by an enzyme electrode method with a Biosen 5030 Autocal glucose-lactate analyzer (EKF Diagnostics, Magdeburg, Germany). Height and weight were measured, and body mass index (BMI) (kg/m^2^) was calculated.

The audiological examinations including pure tone audiometry (PTA) and distortion product otoacoustic emission (DPOAE) test were performed. The average of the hearing levels at frequencies of 0.5, 1.0, 2.0, and 4.0 kHz was calculated as the better ear hearing level (BEHL_0.5–4.0kHz_). The degree of hearing loss was classified as normal (0-25 dB), mild (26-40 dB), moderate (41-60 dB), severe (61-80 dB), or profound (≥81 dB) according to the World Health Organization-International Classification of Impairments, Disabilities and Handicaps (WHO-ICIDH) standard [17]. The DPOAE was accepted as signal-to-noise ratios exceeding 6 dB [18]. Pure-tone averages at the frequency range of 0.5 to 2.0 and 4.0 to 8.0 kHz and average DPOAE signal-to-noise ratios (SNRs) in the right ear at the f2 frequencies of 1.0 to 3.0 kHz and 4.0 to 8.0 kHz were calculated.

Magnetic resonance imaging (MRI) was performed on a 3.0-T Verio Siemens scanner (Erlangen, Germany). We studied patients by axial T1-weighted scan, axial T2-weighted imaging, fluid-attenuated inversion recovery (FLAIR), and diffusion-weighted imaging (DWI).

### 2.4. Mitochondrial Function Evaluation in PBMCs among the Members of F1957

#### 2.4.1. Sample Preparation

Mitochondrial function evaluation was performed as previously described [19]. A total of 4 ml venous blood samples from each individual in F1957 were collected into vacuum tubes containing EDTA. The blood samples were diluted with additional 4 ml phosphate-buffered saline (PBS) and then isolated by Ficoll-Hypaque (Axis-Shield Diagnostics Ltd., Norway). Cells were collected after centrifugation (400g, 30 minutes) and then washed twice with PBS. Finally, sediments were resuspended in RPMI-1640 culture medium supplemented with 10% fetal bovine serum, 100 units/mL penicillin, and 100 g/mL streptomycin for subsequent measurements.

#### 2.4.2. ATP Measurements

Intracellular ATP levels were measured by the ATP bioluminescent assay kit (Beyotime, China) according to the manufacturer's instructions. The BCA assay was utilized to measure the protein concentration of the PBMCs, and the ATP level was finally normalized to the protein content.

#### 2.4.3. Intracellular ROS Measurements

Intracellular ROS production was measured by the reactive oxygen species assay kit (Beyotime, China) according to the manufacturer's instructions. 1 × 10^6^ PBMCs were incubated with 10 *μ*mol/l 2′7′-dichlorodihydrofluorescein diacetate (DCFH-DA) at 37°C for 20 min. Nonfluorescent DCFH-DA can be intracellularly oxidized to the fluorescent compound 2,7-dichlorofluorescein (DCF) by ROS production. The cellular fluorescent changes were finally observed under a fluorescence microscope (Nikon Ti-U, Tokyo, Japan), and the fluorescence intensities were measured by ImageJ.

#### 2.4.4. Mitochondrial Membrane Potential (ΔΨm) Measurements

The mitochondrial membrane potential assay kit (JC-1, Beyotime, China) was used to measure ΔΨm. Briefly, PBMCs were incubated with JC-1 at 37°C for 20 min. In cells with high ΔΨm, JC-1 can accumulate in the mitochondrial matrix showing red fluorescence aggregates. In cells with low ΔΨm, JC-1 remains a monomer showing green fluorescence. Fluorescent changes were observed under a fluorescence microscope (Nikon Ti-U, Tokyo, Japan), and fluorescent images for JC-1 monomers and aggregates were obtained at Ex/Em wavelengths of 490/530 nm and 525/590 nm, respectively. The ratio of red fluorescence and green fluorescence was calculated to represent the level of ΔΨm. A low red : green ratio indicated decreased ΔΨm. All fluorescence intensities were measured by ImageJ.

### 2.5. Whole mtDNA Sequencing in Peripheral Blood among the Members of F1957

Briefly, 500 ng of genomic blood DNA in 100 *μ*l of TE was fragmented to a pool (150-200 bp) by Bioruptor Pico (Diagenode, Belgium), and then adapters (Invitrogen, USA) were ligated to each end of the fragments. The adapter-ligated templates were purified by the Agencourt AMPure XP beads (Beckman, USA). The purified DNA was amplified by ligation-mediated polymerase chain reaction and then purified and hybridized to a mitochondrial genome panel (iGeneTech, China) for enrichment. The target genomic region in the panel included the whole mitochondrial genomes. The hybridized fragments were bound to Streptavidin Dynabeads (Invitrogen, USA) and washed with stringent buffers (iGeneTech, China). The captured products were quantified with the Qubit dsDNA HS Assay Kit (Invitrogen, USA). Paired-end sequencing, which allowed 150 bases from both ends of the fragment for targeted libraries to be read, was performed using Illumina HiSeq Xten instrumentation (Illumina, San Diego, CA) [20].

### 2.6. Statistical Analysis

All statistical analyses were carried out by SPSS version 16 (SPSS Inc., Chicago, IL, USA). Data were represented as the means ± SEM. Student's unpaired two-tailed *t*-test was performed to evaluate the difference between the m.3243A>G carriers and controls. A *P* value < 0.05 was considered statistically significant.

## 3. Results

### 3.1. m.3243A>G Heteroplasmy Analysis of Peripheral Blood, Saliva, and Urine Sediment among 10 Pedigrees

**Figure 1:**
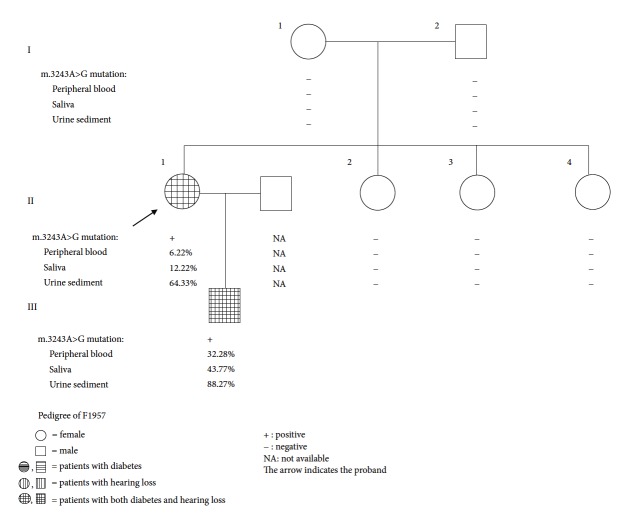
Pedigree of F1957.

The heteroplasmy levels of the m.3243A>G mutation were detected in 31 individuals from 10 unrelated pedigrees by pyrosequencing (Table S1). The average heteroplasmy levels of the m.3243A>G mutation in peripheral blood, saliva, and urine sediment were 17.58 ± 11.19%, 23.62 ± 13.23%, and 59.05 ± 22.71%, respectively. All of the m.3243A>G mutation was inherited matrilineally except for the pedigree of F1957 (Figure 1). The proband from the family 1957 (F1957-II-1) showed heteroplasmy levels of 7.50%, 12.22%, and 64.33% in peripheral blood, saliva, and urine sediment, respectively. The mutation was also detected in her son (F1957-III-1) with higher levels of heteroplasmy (34.16%, 43.77%, and 88.27% in peripheral blood, saliva, and urine sediment, respectively). However, the m.3243A>G mutation was not detected in her parents (F1957-I-1 and F1957-I-2) and siblings (F1957-II-2, F1957-II-3, and F1957-II-4). Parentage identification by microsatellite DNA technology confirmed the genetic relationship between the proband and her parents (data not shown), indicating a de novo m.3243A>G mutation that occurred in the proband.

### 3.2. Whole mtDNA Sequencing in Peripheral Blood among the Members of F1957

The whole mtDNA sequencing analysis of peripheral blood was performed within F1957 including the proband (F1957-II-1) and her son (F1957-III-1), her mother (F1957-I-1), her father (F1957-I-1), and her sister (F1957-II-3). As shown in Table 1, in addition to the m.3243A>G mutation, we found another de novo variation, m.16093T>C in MT-CR (control region), with heteroplasmy levels of 1.57% in the proband. Her son also harbored the m.16093T>C mutation with a higher heteroplasmy level of 8.54%. The ratios of heteroplasmy levels between m.3243A>G and m.16093T>C in the proband and her son were almost same (3.96 and 3.78, respectively), indicating that these two point mutations may spontaneously occur at the same time. These mtDNA point mutations were absent in other family members.

### 3.3. Clinical Evaluation in F1957

We performed a systematical clinical evaluation within the pedigree of F1957. As summarized in Table 2, the proband (F1957-II-1) has been treated with insulin (40 U/day) for 20 years since she was first diagnosed with diabetes at the age of 40. Similarly, her son (F1957-III-1) also has been requiring insulin (30 U/day) therapy for 5 years since he was diagnosed with diabetes at the age of 26. The HbA1c levels of the proband and her son are 6.4% and 7.3%, respectively. The evaluation of serum concentrations of fasting and 2 h postprandial C-peptide suggested that the function of pancreatic *β* cells was not obviously exhausted in both patients. However, elevated lactate levels in blood were found in both the proband and her son (1.97 and 4.17 mmol/l, respectively). In addition, her son also had a history of severe nausea and vomiting. For the hearing loss test, analysis of PTA indices identified mild bilateral sensorineural hearing loss (SNHL) in both of them. The audiograms also showed that hearing levels at high frequencies (4.0~8.0 kHz) were more severely affected in both ears. The DPOAE test showed impaired acceptable responses at high frequencies (4.0~8.0 kHz) in the location of the cochlea with respect to other family members, indicating functional impairment of outer hair cells in the cochlea. As for the evaluation of brain MRI, both the proband and her son showed hyperintensity on T2-weighted images of typical lesions such as bilateral frontal lobe, parietal lobe, and periventricular lesions, indicating partial cerebral ischemia. In addition, brainstem high-signal lesions on T2-weighted images and both cerebral atrophy and cerebellar atrophy were also found in the proband. Other features of MELAS syndrome including migrainous headache, seizures, mental retardation and stroke-like episodes, and lactic acidosis were not found in both of the patients.

All the other family members including the proband's mother and siblings are healthy without clinical manifestation of diabetes and hearing loss or any other mitochondrial disease.

### 3.4. Mitochondrial Function Evaluation in PBMCs of F1957

#### 3.4.1. Increase in ROS Production

**Figure 2:**
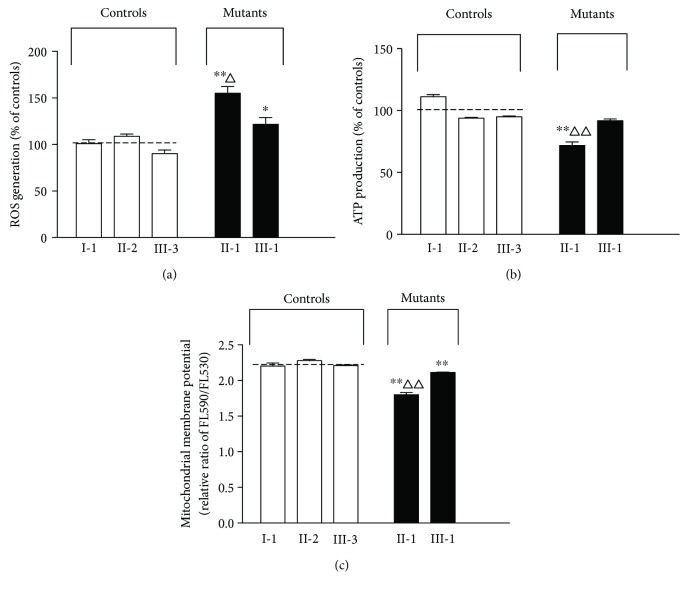
Evaluation of mitochondrial function in PBMCs among individuals from F1957. (a) Intracellular ROS generation in controls and mutants. ROS levels were expressed as relative DCF intensity of controls (%). (b) ATP production in controls and mutants. ATP levels in mutants were normalized to the mean value of controls (%). (c) Mitochondrial membrane potential (ΔΨm) levels of each individual were measured by a fluorescence probe JC-1 assay system. The relative ratio of FL590/FL530 geometric mean was calculated to reflect the levels of ΔΨm. Three to four determinations were made for each individual. Data were expressed as the mean ± SEM. The dash line represents the mean for the group of controls. Compared with control group, ^∗^ *P* < 0.05, ^∗∗^ *P* < 0.01; compared with III-1, ^△^ *P* < 0.05, ^△△^ *P* < 0.01.

The intracellular ROS production of PBMCs was measured by a DCF fluorescence microscope. As shown in Figure 2(a), the levels of the ROS generation in PBMCs derived from the proband (F1957-II-1) and her son (F1957-III-1) were increased by 55.1% (*P* < 0.01) and 21.8% (*P* < 0.05) compared with the mean value measured in her maternal relatives (F1957-I-1, F1957-II-2, and F1957-II-3), respectively. In particular, the proband showed a higher ROS level than her son (*P* < 0.05).

#### 3.4.2. Reduced Level in Mitochondrial ATP Production

To determine whether the capacity of oxidative phosphorylation in PBMCs varies among family members, the levels of cellular and mitochondrial ATP were measured by a luciferin/luciferase assay. As depicted in Figure 2(b), the levels of ATP production in the proband (F1957-II-1) were decreased by 28.2% (*P* < 0.01) compared with the mean value measured in other family members (F1957-I-1, F1957-I-II-2, and F1957-I-II-3). However, her son (F1957-III-1) did not reach the significance compared with controls (*P* > 0.05).

#### 3.4.3. Decrease in the ΔΨm Level

To reflect the bioenergetic parameters including respiratory rate, ATP synthesis, electron transport, and proton leaks, the ΔΨm level was measured by a fluorescence probe JC-1 assay system. As depicted in Figure 2(c), the ΔΨm levels in the proband (F1957-II-1) and her son (F1957-III-1) were significantly lower than those in other family members (1.801 ± 0.056 vs. 2.230 ± 0.041 and 2.113 ± 0.009 vs. 2.230 ± 0.041, respectively, *P* < 0.01), indicating a decreased ΔΨm level. Moreover, the proband showed a lower ΔΨm level than her son (*P* < 0.01).

## 4. Discussion

In our study, we used pyrosequencing to analyze the heteroplasmy levels of the m.3243A>G mutation in peripheral blood, saliva, and urine sediment, which enabled the mitochondrial heteroplasmy level to be accurately quantified from 0% to 100% [16]. Our study identified that the m.3243A>G mutation in the proband (F1957-II-1) spontaneously occurred without maternal inheritance.

The proband (F1957-II-1) and her son (F1957-III-1) both manifested diabetes with mild bilateral SNHL, both of which had typical clinical characteristics of MIDD. Besides, the proband also showed abnormal MRI of brain atrophy which was often found in MIDD patients [3, 21]. The manifestations of hearing loss and brain atrophy are mainly due to the fact that the cochlea and brain are both metabolically active organs and patients with MIDD are easier to be affected because of cellular ATP deficiency [22]. In addition, all of her maternal relatives who do not harbor the mutation were clinically healthy without any manifestation of diabetes and hearing loss or any other mitochondrial disease.

The analysis of mitochondrial function in the PBMCs revealed a higher level of ROS production and lower levels of mitochondrial ATP and ΔΨm in the proband and her son compared with their family members, indicating that impaired mitochondrial functions were found even in easily obtainable circulating PBMCs in these spontaneous mutant carriers. Moreover, the proband showed a relatively worse mitochondrial function including all of the ROS, ATP, and ΔΨm when compared to her son, probably due to the influence of aging on mitochondrial function.

The whole mtDNA sequencing of peripheral blood in F1957 identified another heteroplasmic substitution at m.16093T>C in both the proband and her son, which has not ever been reported in MIDD or MELAS syndromes. The position 16093 of mtDNA is involved in a noncoding sequence located in hypervariable region 1 (HV1) that includes the extended termination-associated sequence 2 domain (ETAS2) from nt 16081 to 16138, a functional location involved in the regulation of mtDNA replication and transcription. It is noteworthy that the sequence variation in nt 16040–16188 segment of HV1 was found to be associated with cyclic vomiting syndrome (CVS) and migraine headache in previous studies [23]. Interestingly, the son of the proband also had a history of severe nausea and vomiting. We suspect that the substitution of m.16093T>G together with the m.3243A>G mutation is one of the factors that modify the clinical features and affect the mitochondrial function in the patient F1957-III-1.

As for the inheritance of mtDNA mutation-related diseases, mtDNA can only be derived from the mother [24]. In previous studies, almost all matrilineal relatives involved in the MIDD-like mitochondrial disorder were found to carry the same mutation in their various original tissue cells, even though at low levels [25, 26]. However, a few reports also found that the de novo mutation of the m.3243A>G occurs at times [5, 7]. Harihara et al. found a pair of twins carrying the m.3243A>G mutation among 32 families of identical twins, though the twins did not show any clinical abnormality at the age of 12 [5]. In our case, all of the evidences support that de novo m.3243A>G together with another mtDNA point mutation occurred at the same time and the mitochondrial function of PBMCs was impaired in the proband. Our study indicates that proper genetic screening is necessary for patients manifesting typical MIDD syndrome even without a family history.

Unlike nuclear DNA (nDNA), mtDNA is easy to accumulate missense mutations for lacking a DNA repair system [27]. As for the major cause of mtDNA mutation, many reports suggested that oxidative damage during aging and environmental factors like smoking and chemicals could accelerate the process of mutation aggregation [28–30]. It should be noted that the proband's mother (F1957-I-1) had an experience of working at a plastic product factory during the period of her conception. The presence of the m.3243A>G mutation in the blood cells, buccal mucosa, and urine sediments and the abnormal function of the pancreas and cochlea which originates from different germ layers in the proband suggest that the mutation might have existed in the very early stage like the embryonic stage or germ cell stage.

In conclusion, we found a typical clinical change of the MIDD syndrome and impaired mitochondrial function in PBMCs in the spontaneous m.3243A>G mutation carriers. Our study also suggests that the de novo m.16093T>G substitution can occur together with m.3243A>G, and the additional mtDNA missense mutations may constitute risk factors for clinical features and mitochondrial disease further development.

## Figures and Tables

**Table 1:** Novel mtDNA substitutions detected in F1957 by whole mtDNA sequencing.

Person	Sex	Age	m.3243A>G		m.16093T>C		Ratio^#^
			Het%	Location	Het%	Location	
F1957-II-1 (proband)	Female	60	6.22	MT-TL1	1.57	MT-CR	3.96
F1957-III-1	Male	31	32.28	MT-TL1	8.54	MT-CR	3.78
F1957-I-1	Female	80	—		—		
F1957-I-2	Male	83	—		—		
F1957-II-3	Female	57	—		—		

Het: heteroplasmy; ^#^ratio of m.3243A>G (Het%) to m.16093T>C (Het%).

**Table 2:** Clinical and biochemical characteristics among the pedigrees of F1957.

	II-1	III-1	I-1	I-2	II-2	II-3	II-4
m.3243A>G mutation	+	+	-	-	-	-	-
Sex (M/F)	F	M	F	M	F	F	F
Age (years)	60	31	80	83	57	55	51
BMI (kg/m^2^)	19.5	18.5	15.7	21.7	21.0	21.0	15.6
Blood lactic acid (mmol/l)	1.97	4.17	1.58	NA	0.82	0.92	1.07
Diabetes							
Onset age of diabetes (years)	40	26	-	-	-	-	-
HbA1C (%)	6.4	7.3	5.4	5.7	5.5	5.5	5.7
GA (%)	14.6	21.2	15.3	13.4	12.5	13.2	15.1
Fasting plasma glucose (mmol/l)	6.78	10.43	5.20	5.92	5.36	4.74	5.56
2 h postprandial plasma glucose (mmol/l)	10.72	15.66	6.99	NA	6.10	3.92	5.01
Fasting C-peptide (ng/ml)	1.01	2.22	1.02	1.75	2.01	1.66	1.62
2 h postprandial C-peptide (ng/mL)	2.27	3.22	9.39	NA	8.85	5.00	10.41
Treatment	Insulin (40 U/day)	Insulin (30 U/day)	-	-	-	-	-
SNHL							
Symptoms of deafness	-	-	-	-	-	-	-
Pure-tone averages							
BEHL_0.5-4.0kHz_	30 (mild +)	31.25 (mild +)	22.50 (-)	NA	13.75 (-)	10.00 (-)	NA
0.5-2.0 kHz	28.33	27.50	23.33	NA	13.33	15.00	NA
4.0-8.0 kHz	57.50	52.50	30.00	NA	33.75	15.00	NA
DPOAE SNR averages in the right ear							
1.0-3.0 kHz	3.18	6.58	NA	NA	12.9	9.15	NA
4.0-8.0 kHz	−1.63	−1.23	NA	NA	5.63	6.27	NA
Brain MRI							
High-signal lesions on T2-weighted images	Bilateral frontal lobe, periventricular, brainstem	Bilateral frontal lobe, left parietal lesion	NA	NA	NA	-	NA
Brain atrophy	+	-	NA	NA	NA	-	NA
Atypical clinical features							
Nausea and vomiting	-	+	-	-	-	-	-

BMI: body mass index; HbA1c: glycated hemoglobin; GA: glycated albumin; GAD-Ab: glutamic acid decarboxylase; IA2-Ab: islet antigen 2; ACR: urinary albumin-to-creatinine ratio; SNHL: sensorineural hearing loss; BEHL: better ear hearing level; DPOAE: distortion product otoacoustic emission; -: negative; NA: not available.

## Data Availability

Data are included in this published article and its supplementary information files. We can provide detailed data upon reasonable request.

## Supplementary Materials

Supplementary Materials Table S1: m.3243A>G heteroplasmy among individuals from 10 pedigrees with MIDD.
