# Supplementary material for: De Novo Mutation of m.3243A>G together with m.16093T>C Associated with Atypical Clinical Features in a Pedigree with MIDD Syndrome
Source: J Diabetes Res. 2019 Apr 4;2019:5184647. doi: 10.1155/2019/5184647 (PMC6476128; doi:10.1155/2019/5184647)
Supplement: Supplementary Materials — Table S1: m.3243A>G heteroplasmy among individuals from 10 pedigrees with MIDD. [file 5184647.f1.pdf]

Table S1. M.3243A>G Heteroplasmy among Individuals from 10 Pedigrees of MIDD

Pedigree	Person	Sex (F/M)	Age (years)	Heteroplasmy, %		
				Leukocytes	Saliva	Urine Sediment
F1921	F1921- Ⅰ-1	F	55	19.43	22.69	63.14
	F1921- Ⅱ-1	F	31	35.35	36.54	63.14
	F1921- Ⅱ-2	F	32	22.69	28.33	38.93
F1955	F1955- Ⅰ-1	M	74	0.84	3.20	15.23
F1932	F1932- Ⅰ-1	F	60	10.29	13.21	32.98
	F1932- Ⅱ-1	F	32	14.21	19.43	60.75
	F1932- Ⅱ-2	M	30	32.98	43.77	84.33
F1958	F1958- Ⅰ-1	F	48	11.25	15.23	59.55
	F1958- Ⅰ-2	F	45	26.05	29.48	69.01
	F1958- Ⅱ-1	F	21	3.20	3.20	5.73
	F1958- Ⅱ-2	M	24	44.98	55.93	90.16
F1949	F1949- Ⅰ-1	M	63	4.87	10.29	49.85
	F1949- Ⅰ-2	M	56	13.21	16.26	65.51
	F1949- Ⅰ-3	M	50	18.36	29.48	84.33
	F1949- Ⅱ-1	M	45	-	-	-
	F1949- Ⅱ-2	M	30	-	-	-
F1956	F1956- Ⅰ-1	M	72	5.73	8.41	37.73
F1918	F1918- Ⅰ-1	M	59	19.43	32.98	75.82
	F1918- Ⅱ-1	M	51	12.22	22.69	73.59
	F1918- Ⅱ-2	M	45	16.26	31.81	80.17
F1932	F1932- Ⅰ-1	M	57	12.22	14.21	34.16
F1957	F1957- Ⅰ-1	F	80	-	-	-
	F1956- Ⅰ-2	M	83	-	-	-
	F1957- Ⅱ-1	F	60	6.22	12.22	64.33
	F1957- Ⅱ-2	F	57	-	-	-
	F1957- Ⅱ-3	F	55	-	-	-
	F1957- Ⅱ-4	F	51	-	-	-
	F1957-Ⅲ-1	M	31	32.28	43.77	88.27
F1989	F1989- Ⅰ-1	F	48	12.22	23.80	71.32
	F1989- Ⅱ-1	F	44	27.18	29.48	69.01
	F1989- Ⅱ-2	F	28	17.30	20.50	40.14

-：negative
